# Myocardial first-pass perfusion cardiovascular magnetic resonance: history, theory, and current state of the art

**DOI:** 10.1186/1532-429X-10-18

**Published:** 2008-04-28

**Authors:** Bernhard L Gerber, Subha V Raman, Krishna Nayak, Frederick H Epstein, Pedro Ferreira, Leon Axel, Dara L Kraitchman

**Affiliations:** 1 Department of Cardiovascular Diseases, Cliniques Universitaires St Luc, Université Catholique de Louvain, Brussels, Belgium; 2 Davis Heart and Lung Research Institute, Division of Cardiovascular Medicine, Ohio State University, Columbus, OH, USA; 3 Ming Hsieh Department of Electrical Engineering, University of Southern California, Los Angeles, CA, USA; 4 Departments of Radiology and Biomedical Engineering, University of Virginia, Charlottesville, VA, USA; 5 National Heart and Lung Institute, Imperial College, London, UK; 6 Department of Radiology, New York University Medical Center, New York, NY, USA; 7 Russell H. Morgan Department of Radiology and Radiological Science, The Johns Hopkins University, School of Medicine, Baltimore, MD, USA

## Abstract

In less than two decades, first-pass perfusion cardiovascular magnetic resonance (CMR) has undergone a wide range of changes with the development and availability of improved hardware, software, and contrast agents, in concert with a better understanding of the mechanisms of contrast enhancement. The following review provides a perspective of the historical development of first-pass CMR, the developments in pulse sequence design and contrast agents, the relevant animal models used in early preclinical studies, the mechanism of artifacts, the differences between 1.5T and 3T scanning, and the relevant clinical applications and protocols. This comprehensive overview includes a summary of the past clinical performance of first-pass perfusion CMR and current clinical applications using state-of-the-art methodologies.

## Introduction

The clinical assessment of myocardial perfusion plays a central role in the diagnosis, management, and prognosis of ischemic heart disease patients. Whereas X-ray angiography demonstrates the patency of the coronary arteries, perfusion imaging detects the downstream microvascular blood flow within the myocardium. Single photon emission computed tomographic (SPECT) imaging of myocardial perfusion with ^201^TI or ^99m^Tc-labeled agents is an established clinical standard [1-4], but has relatively poor spatial resolution, suffers from soft tissue attenuation artifacts, is not quantitative, and delivers a significant dose of ionizing radiation[1]. Positron emission tomography (PET) overcomes some of the limitations of SPECT; however, availability is restricted to sites with a cyclotron for supplying short-half-life radiotracers. Echocardiography-based perfusion is another technique but has limited acoustic windows, low spatial resolution, and concerns exist about contrast agent administration.

Myocardial perfusion imaging by first-pass contrast-enhanced cardiovascular magnetic resonance (CMR) was introduced in 1990, when Atkinson et al. first used inversion-recovery gradient-echo imaging after injection of a bolus of Gd-DTPA to observe contrast agent transit through the cardiac chambers and myocardium[5]. Subsequently, the technique has undergone continuous technical development, experimental validation, and clinical evaluation. Technical developments have occurred in the areas of MR hardware (gradient systems, magnetic field strength, radiofrequency coil arrays), pulse sequence design, new contrast agents, and perfusion analysis methods. Evaluation has included preclinical imaging in animal models of ischemic heart disease, as well as clinical studies. While the earliest CMR studies had limitations, such as poor slice coverage and low temporal resolution, recent clinical studies show that CMR now compares quite favorably to SPECT and PET [6-8].

In this article, technical issues related to pulse sequences, contrast agents, and analysis methods are discussed. Both visual interpretation of images and quantitative analysis methods are described. A review of preclinical and clinical evaluation studies is also provided. In addition, practical clinical matters such as safety, patient monitoring, and proper training of personnel are covered. The article also addresses some remaining unanswered questions: Will a field strength of 3T be advantageous compared to 1.5T? Given the expense in time and effort, is quantitative analysis better? Will we eventually understand and resolve the confounding dark rim artifact?

### Contrast agents

There are two major classes of contrast agents used for first-pass perfusion imaging. The extravascular, extracellular class of agents were the first agents approved for clinical use. Due to the small size of these agents, they rapidly diffuse from the vascular space to the extravascular space. While a wide range of agents have been used, including manganese, gadolinium, and dysprosium, gadolinium-based agents are the only clinically approved agents, albeit primarily for brain and body imaging.

Due to its seven unpaired electrons, gadolinium is one of the most effective paramagnetic agents[9]. However, due to the extreme toxicity of free gadolinium, gadolinium is always chelated. Paramagnetic contrast agents alter the local magnetic field and thereby enhance the relaxation rate of water protons in close proximity to the contrast agent. Thus, intravenous delivery of low doses of paramagnetic agents results in enhanced signal in T1-weighted images in the tissues that are perfused. For low doses of paramagnetic agents, an approximately linear relationship between the image intensity and contrast agent concentration exists[10]. For cardiovascular imaging, the dosage is often not kept low enough for this linearity assumption to apply. However, the dosage is sufficient low such that T2 and T2* effects do not predominate throughout the scan. Thus, perfused tissue appears brighter during the first pass of the gadolinium-based contrast agent[5]. While extravascular, extracellular contrast agents are excluded from the intracellular space, water can exchange between the intravascular and extracellular and intracellular spaces. Thus, the effective concentration of contrast agent in the tissue will be lower than the "true" intravascular and extracellular concentrations.

The second major class of first-pass perfusion agents, which are also primarily gadolinium-based, are the intravascular agents. Diffusion from the vascular space is inhibited by the large size of these agents. Their large size also effectively decreases tumbling rates, resulting in a higher relaxivity than the extravascular, extracellular agents. One intravascular agent, trisodium- [2-(R)-[(4,4-diphenylcyclohexyl) phosphono oxymethyl]-diethylenetriaminepentaacetato) (aquo) gadolinium (III) (Vasovist, Schering AG) is currently approved for use in Europe. This agent reversibly binds to albumin in the plasma, effectively resulting in a macromolecular, intravascular MR contrast agent. While intravascular gadolinium-based contrast agents were initially developed for MR angiography, several groups have identified advantages of these agents for first-pass perfusion imaging [11-14].

### Animal cardiovascular disease models for perfusion imaging

To test new contrast agents and validate new imaging sequences, phantoms and animal models of human cardiovascular disease are often employed. First-pass perfusion imaging of the heart using contrast agents on clinical scanners has only been performed in larger animals, e.g., dogs, sheep, and pigs, due to their lower heart rates and larger cardiac size than murine models.

When considering animal models for human cardiovascular disease, one of the biggest concerns is whether the degree of collateral circulation mimics man. Maxwell et al. demonstrated that dogs and cats possess the greatest coronary collateral flow after acute ischemia, with pigs possessing the least, and rats, rabbits, and sheep intermediate between dogs and pigs[15]. There are also gradients in perfusion across the heart wall, such that the high subepicardial flow in dogs leads to non-transmural, subendocardial infarcts with acute coronary occlusion, whereas pigs typically have lower epicardial flow and develop transmural infarctions[16]. On the other hand, rats typically develop non-transmural infarcts that do not extend to the subendocardium. The largest determinant of whether tissue is reversibly or irreversibly damaged is the duration of occlusion. The earliest studies of contrast-enhanced, first-pass perfusion imaging examined acute infarction animal models that were either reperfused or permanently occluded. Subsequently, animal models of perfusion abnormalities without infarction have relied on models that either limit epicardial artery blood flow or study the effects of short-term coronary artery occlusion followed by reperfusion [14,17,18]. Another approach is to study perfusion pathways that develop with collateral vessel formation in acute angina. Extensive studies in swine, who lack native collaterals, have used an ameroid occluder, placed around coronary artery branches [19]. Thus, animal models may provide a platform for validation of perfusion image pulse sequences and analysis techniques, but the limitations of these models must be understood for translation to the clinical realm.

### Pulse sequences

**Figure 1:**
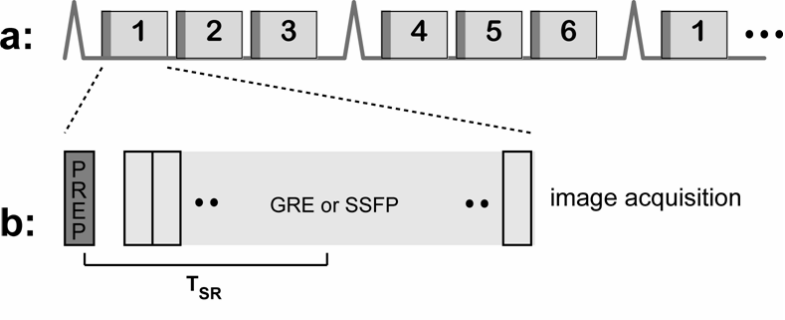
Typical first-pass perfusion pulse sequence. (A) Acquisitions are cardiac gated using ECG signals. Multiple slices are acquired consecutively (shown: 6 slices every 2 R-R intervals). This is repeated continuously during the first-pass and washout of the contrast agent. (B) T1 contrast is generated using a saturation pulse (dark gray) followed by fast imaging of each slice (light gray) using gradient echo, gradient echo-planar, or steady state free precession acquisitions. Image contrast is primarily determined by the saturation recovery time, labeled TSR.

The imaging pulse sequence is critical in determining the image contrast, spatial resolution, and coverage and influences the potential for quantification and/or the presence of artifacts. This section reviews the basics; a more comprehensive review of perfusion imaging sequences was recently published by Kellman and Arai [20]. The basic requirements of a first-pass myocardial perfusion imaging pulse sequence are to provide: 1) strong T1 contrast; 2) coverage of relevant myocardial segments; and 3) adequate spatial resolution. Figure 1 contains a schematic of a typical first-pass imaging sequence.

To provide T1 contrast, inversion recovery (IR) was the first contrast preparation to be used, but was limited to one or a few slices and proved sensitive to heart rate variation. Saturation recovery (SR) has emerged as the current standard because contrast is theoretically independent of the magnetization "history," e.g., heart rate and previous acquisitions, and a higher numbers of slices (such as six to eight) can be provided with interleaved acquisitions. SR is typically implemented using a non-selective 90 degree radiofrequency (RF) excitation followed by a gradient crusher. This approach can be sensitive to variations in the transmitted RF field[21], which has recently led to the use of pulse trains, composite RF pulses, and B1-insensitive rotation pulses (e.g., BIR-4), that provide more uniform saturation in practice, albeit with increased RF heating.

Coverage of relevant myocardial segments is achieved using an interleaved acquisition of arbitrarily oriented slices. In Figure 1, six slices are acquired with a temporal resolution of 2 R-R intervals. This could, for example, include six short-axis slices or four short-axis slices plus two-chamber and four-chamber long-axis slices. Note that the slices have different cardiac phases, but each slice is acquired repeatedly at the same cardiac phase. Slices obtained during systole have the advantage of increased myocardial thickness (i.e., more pixels across the wall), while those obtained during the relatively stable cardiac phases (e.g. mid-diastole and end-systole) have the advantage of reduced motion artifacts. When the patient's heart rate is elevated, the number of slices per R-R may need to be reduced. With the goal of identifying large-vessel CAD, three to six slices are routinely used. With the goal of characterizing the extent of disease, there have been recent attempts at providing contiguous whole-heart coverage (similar to what SPECT and PET provide) using either a large number of 2D slices or native 3D encoding [22].

In multislice perfusion imaging, the slice thickness is typically 5 to 10 mm, and in-plane resolution is typically 1.5 to 3 mm. Depending on the resolution, field-of-view (FOV), and use of acceleration, image acquisition of each slice can take on the order of 50 – 200 ms. This requires an ultra-fast acquisition, usually based on gradient echo (GRE), gradient echo-planar (GRE-EPI)[23,24], or balanced steady-state free precession (SSFP). Parallel imaging [25-27] is also widely used as a means for accelerating image acquisition. SSFP provides a higher signal-to-noise ratio compared to GRE, but can suffer from off-resonance artifacts that limit the repetition time (TR). GRE is the basis of today's most robust protocols at 1.5T and 3T, while SSFP has produced promising results at 1.5T [28].

Perfusion quantification is an active area of investigation (see "Image Analysis" section) that can require a modified acquisition. For example, measurements of regional myocardial blood flow (in ml/g/min) can be made using time-intensity curve deconvolution with a measured arterial input function (AIF), usually from the left ventricular cavity or ascending aorta[29,30]. Measurement of the true AIF is affected by short T1s and T2* effects[31] that occur with high contrast agent concentrations during bolus contrast agent passage in the blood pool. This has led to two imaging strategies: 1) the "dual-bolus" method[32,33], which involves AIF measurement during a separate low-dose injection (1/10^th ^or 1/20^th ^the dose) prior to the full-dose injection used for the measurement of myocardial enhancement and 2) the "dual-sequence" method [34-36], which replaces one of the imaging slices with an AIF measurement slice that employs an acquisition that avoids the saturation and T2* effects at high concentrations.

### Artifacts

Several artifacts can occur in first-pass perfusion imaging that must be recognized in order to correctly interpret the images qualitatively. The most prominent and troublesome artifact is the dark rim artifact (DRA). Recognizing the causes of the DRA and other artifacts can aid in adjusting the imaging parameters to minimize the consequences of the artifacts.

#### The dark rim artifact

**Figure 2:**
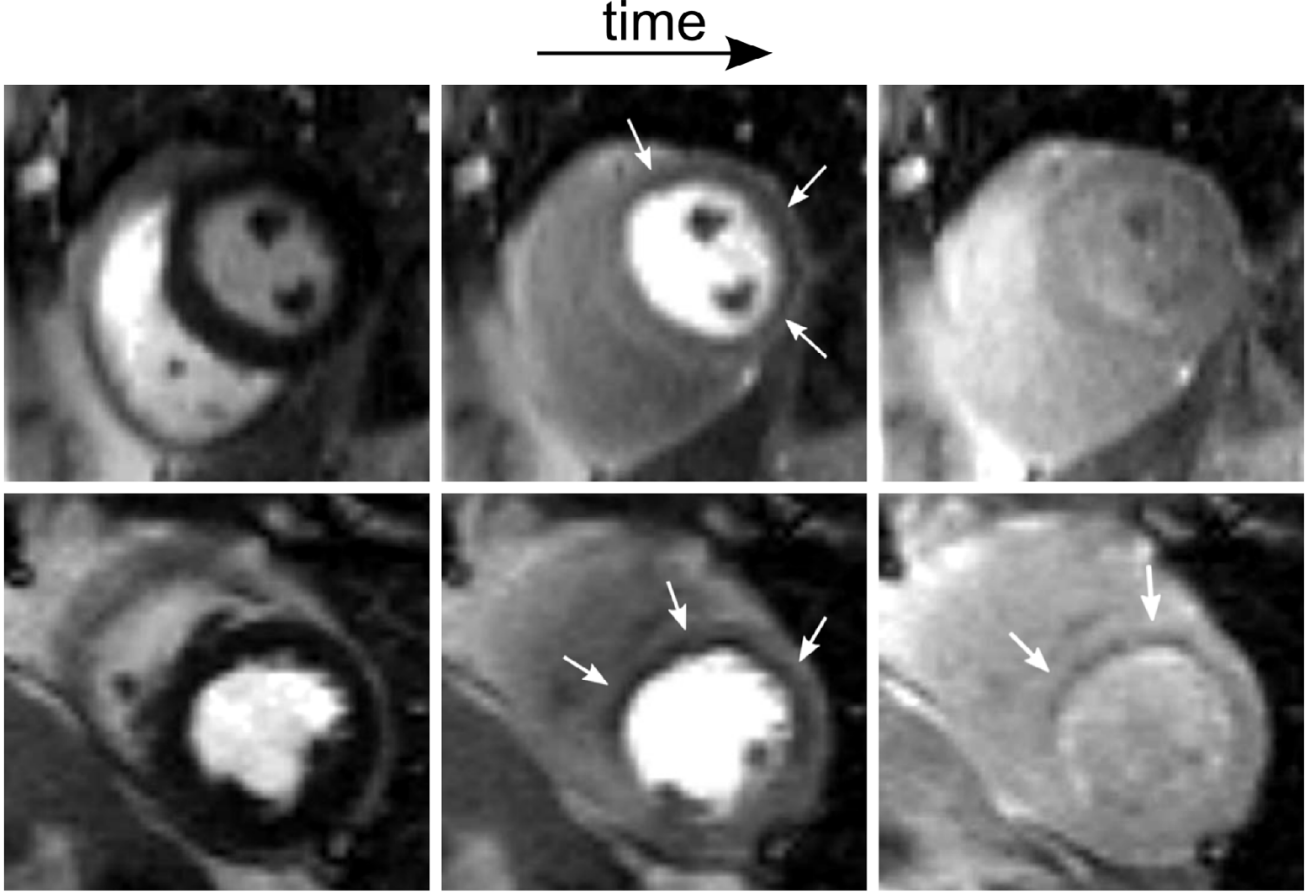
Comparison between a dark rim artifact (DRA, top) and a real perfusion defect (bottom) from two different patients. From left to right are shown the contrast arrival to the left ventricle, the myocardium, and finally recirculation. A DRA artifact usually lasts for a few heartbeats while a real defect tends to be more persistent.

In some perfusion scans, a transient dark rim artifact is visible in the subendocardial layer, which can mimic a hypoperfused area. An experienced observer can differentiate between the DRA and a real perfusion defect by the transient behaviour of the DRA. DRAs normally last for a few heart beats, and vary temporally as the contrast bolus passes through the left ventricle blood pool, while a real perfusion defect tends to be visible for a longer duration of the imaging (Figure 2) [37]. However, a mild perfusion defect which "fills in" quickly is often difficult to identify in the presence of artifact. Another property of this artifact is that the signal intensity drops below the baseline signal, i.e., below the signal before the contrast arrival [38,39]. Although a visual assessment can be used to identify an artifact, a semi-quantitative or quantitative analysis can become complex and erroneously estimate blood flow when DRAs are present. Therefore, DRAs should be avoided whenever possible.

Usually the perfusion sequence is acquired at rest and stress. The DRA may be altered by faster cardiac motion and a more concentrated Gd bolus due to increased cardiac function at stress, leading to increased left ventricular (LV) blood pool signal and more artifact at the margin of the blood pool. However, the rest scan should not be taken as a guarantee against DRA. The effect of residual contrast agent in the second perfusion series should also be considered.

**Figure 3:**
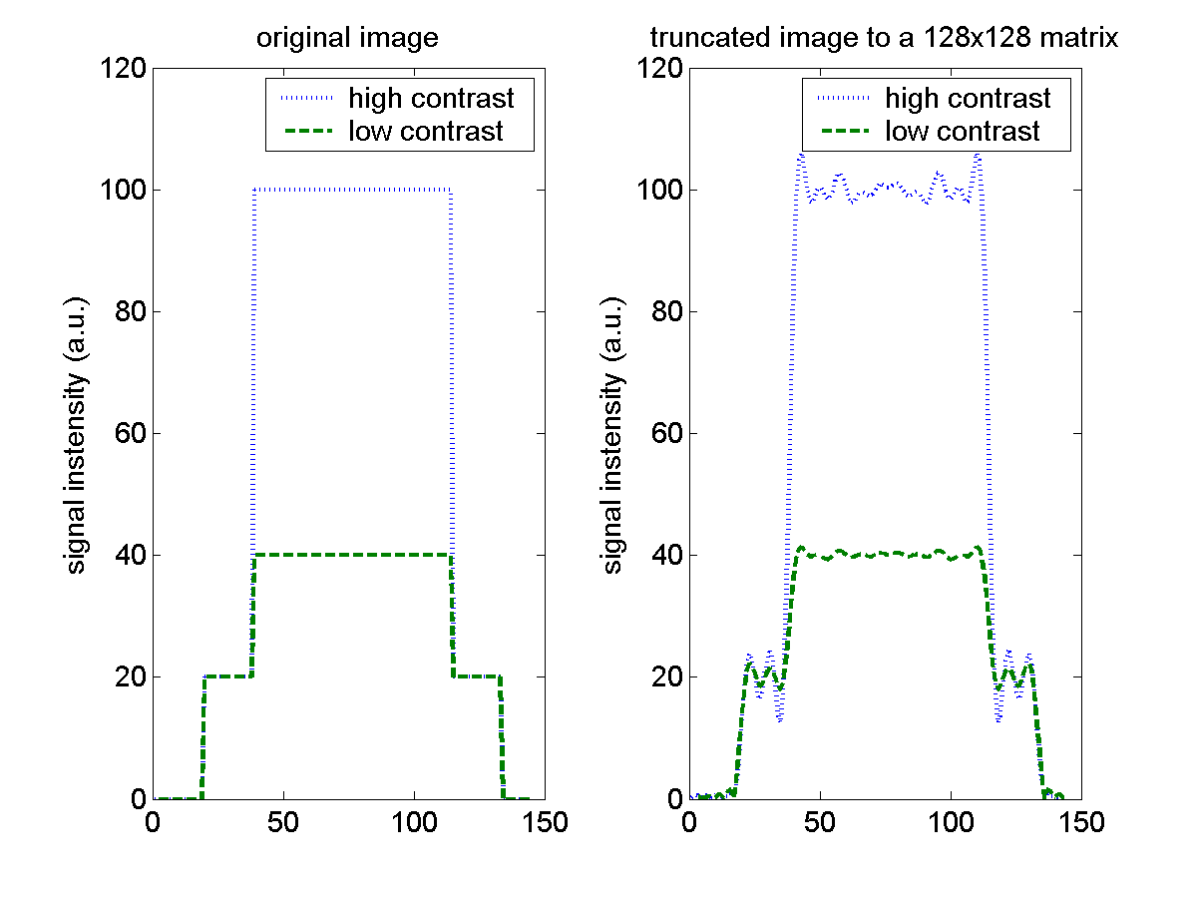
Simulation of a short axis line profile. Comparison between a high and a low contrast (between the LV blood and the myocardium) line profile. Signal oscillations are higher for the high contrast image, as expected from the Gibbs truncation theory. (See Additional file 1.)

Several possible causes have been suggested for DRAs. Di Bella et al [40]. and others have suggested the possibility of the artifact being produced largely by Gibbs ringing in the phase encode direction (direction with the lowest resolution) at the blood-myocardial interface. A Fourier transform of a finite signal has inherent difficulty in representing a discontinuity; therefore, it will not reconstruct sharp edges perfectly and will always have at least a 9% (arising from the Wilbraham-Gibbs constant) signal variation (undershoot and overshoot) near the edge. A DRA caused by Gibbs ringing would be dependent on the contrast between the LV and the myocardium; this could be the reason that the intensity of the artifact varies temporally with the contrast between the blood pool and the myocardium, (Figure 3 and Additional file 1). This explanation is consistent with the higher concentration of the contrast agent in the heart with stress, increasing the Gibbs ringing at the endocardial border. The same effect is expected from a higher injection rate of the contrast agent. Balanced SSFP sequences are the most likely to suffer from Gibbs because they produce a higher signal intensity difference between the blood pool and myocardium. If the spatial resolution is sufficiently low, Gibbs ringing could be severe enough to affect the entire interventricular septum, particularly during diastole. An obvious solution to avoid Gibbs ringing is to increase resolution, sampling larger regions of k-space. However, due to time constraints in cardiac imaging, this may not be possible. Parallel imaging can be used to increase resolution without increasing the duration of each image, but as a trade-off the signal-to-noise ratio (SNR) decreases. Another option to reduce Gibbs ringing is to filter the k-space with a window function, with the trade-off of lowering spatial resolution.

The work done by Arai,[37] Fenchel et al.,[41] and Schreiber et al. [39] attributes magnetic susceptibility, associated with the concentration of Gd-DTPA in the bolus, as a possible cause for the DRA. Increased distortions in the magnetic field around boundaries may cause dephasing in individual voxels and possible signal misregistration due to alterations in the Larmor frequency. Albert et al.[42] studied the mechanism of bulk magnetic susceptibility induced by a contrast agent bolus injection. This study showed that, in an approximation to an infinitely long right cylinder (resembling the LV blood pool and the myocardial wall), the magnetic field of the surroundings of the cylinder is distorted with the passage of a paramagnetic substance inside the cylinder. This effect is also dependent on the orientation of the cylinder in relation to the main magnetic field, B0. Thus, the susceptibility change is dependent on the heart orientation in relation to B0, which will vary from patient to patient. A change of magnetic susceptibility, caused by the arrival of the Gd in the LV blood pool, in the DRA region was measured by Schreiber et al.[39] Gibbs ringing was dismissed due to the particular orientation of the artifact. The artifact was in fact only visible when using a b-SSFP sequence, due to its susceptibility sensitivity that can be avoided by decreasing TR. The use of a high dose of contrast agent will also more likely contribute to field distortion. The increase in the Gd concentration at stress is therefore more likely to produce a susceptibility artifact.

Banding artifacts at tissue boundaries may occur when the object moves between the acquisitions of different phase encode lines and could be another possible origin of the DRA [43]. Parallel imaging can reduce the artifact width by a factor equal to the square root of the acceleration parameter. This study also showed that the direction of the artifact is in the phase encode direction if the motion is in that direction, or is oblique to the frequency encode direction in case of motion in the latter direction. Simulations of the intensity profile of a tissue boundary, modeled as a step function, showed that when sequential ordering of k-space data acquisition is used the oscillations occur in the brighter parts of the image, independent of the motion direction; sequential ordering was preferred to a centric ordering due to lower artifact amplitude [43]. Motion will result in oscillations in the pixel values along the borders of different tissues with different intensities, making it a good contender for a cause of DRA. Again, this artifact correlates with the brightness difference between blood and myocardium. A likely increase of motion artifacts is expected during stress, as discussed above.

Another possible origin for DRA is the non-uniform raw k-space data weighting in the phase-encode direction, i.e., signal variations during the acquisition [20]. Distortions in the point spread function along the phase-encode direction result, due to data acquisition prior to reaching the steady state. A dependency on T1, the time delay between the readout and the saturation pulse, the sequence used, and the flip angle will occur. Because the LV blood pool and the myocardium have different T1s, a DRA can be formed in the boundary between them. A possible way to minimize this artifact is to use an optimized transition to equilibrium like the one used by Hennig et al. [44].

Partial volume effects may also produce a DRA at the border between the LV blood pool and the endocardium. Using an inversion recovery preparation at certain inversion delays, the phases of both tissues can be opposite, resulting in signal cancellation at this boundary. However, this artifact would be expected to appear around the entire border [20,40]. A larger partial volume artifact might appear in the apical slice, where the myocardial wall might not be perpendicular to the imaging plane, with the presence of myocardial wall and the LV blood pool in the same voxel.

The most frequently used pulse sequences for myocardial perfusion studies have been studied to determine which are most prone to DRA. Elkington et al.[45] compared a hybrid-echoplanar imaging sequence with spoiled GRE in patients with CAD and found no significant difference in artifact scoring between the two. Wang et al.[28] compared a b-SSFP sequence with spoiled GRE and segmented EPI in volunteers with no clinical history of CAD. The study found a higher DRA score for the bSSFP sequence when compared to the other two sequences. A similar study using healthy subjects found a higher score for DRA when using a b-SSFP sequence, this time compared to spoiled GRE [41].

Recently, Klem et al. described a method for diagnosis of coronary artery disease (CAD) where adenosine-stress and rest perfusion cardiovascular magnetic resonance (CMR) are combined with late gadolinium enhancement (LGE) CMR [46]. This method particularly implies that perfusion defects detected both at rest and stress combined with no detection of infarcted areas on the late gadolinium enhancement should be interpreted as an DRA.

#### Other artifacts

Although DRAs represent the most serious artifact in myocardial perfusion imaging, several other types of artifacts may also occur. Semiquantitative and quantitative analyses of myocardial perfusion studies assume a perfect magnetisation preparation; qualitative analysis will also improve with a complete saturation. Saturation pulses are, in general, not perfect, due to B0 and B1 field inhomogeneities, leaving regional magnetisation residues that alter the apparent T1 relaxation times and eventually lead to errors in quantifying perfusion in those areas. Kim et al[21] evaluated favourably the use of a B1 Insensitive Rotation (BIR-4) saturation pulse to account for the field distortions. This RF pulse is effective in producing a complete saturation at 1.5T. High field strengths will, very likely, make it even more useful, but with additional specific absorption rate (SAR) concerns.

A common artifact seen in perfusion studies is wrap artifact, where one part of the anatomy might be encoded in the same position as another, creating signal interference. Several solutions exist, such as in-plane rotation of the phase/frequency encode direction or increasing the phase-encode field of view. Parallel imaging techniques can also produce artifacts, particularly spatially variant noise, which is related to the position of the coils on the patient, and aliasing due to errors in the sensitivity estimation of the coils. Chemical shifts and N/2 artifacts can also be problematic for GRE-EPI sequences. Therefore these sequences usually incorporate fat suppression pulses and phase correction reference scans, respectively.

### 1.5T vs. 3T issues

Whole body 3T scanners are increasing in availability, prompting comparison between 1.5T and 3T for CMR. Higher field strengths have the advantage of higher SNR, but there are also a number of possible disadvantages that could lead to artifacts in perfusion imaging. Firstly, both the B0 and B1 field inhomogeneities are increased and greater care has to be taken to reduce the impact of these. Secondly, the T1 is increased and this, coupled with a shorter T2*, could lead to a reduced signal and perfusion contrast. The relaxivity of the commonly used Gd-DTPA is 3.7 mmol/L/s at 3T versus 4.1 mmol/L/s at 1.5T; therefore, the T1 shortening effect of the contrast agent is reduced at 3T. Nevertheless, precontrast T1 is higher for higher fields, making the difference of T1 before and after contrast larger for high fields and enabling an overall better contrast to noise ratio (CNR) for myocardial enhancement [36,47]. However, increases in SAR at 3T can significantly limit any possible SNR gains.

An SNR increase of 109% was obtained by Gutberlet et al. using a T1-weighted segmented EPI with an echo train length of 4 at 3T when compared to 1.5T [48]. Araoz et al. obtained an SNR of 82 ± 26 and 25 ± 8 for 3T and 1.5T, respectively, at peak myocardial enhancement using an interleaved notched saturation recovery gradient echo pulse sequence with an echo train of 4 [49]. At clinical doses of contrast agent, i.e., 0.1 mmol/kg, the SNR increased in the myocardium, but the LV blood pool SNR was similar between 3T and 1.5T, suggesting that an increase in the Gd concentration creates T2* signal loss that negates the increased signal at higher field strengths. Hinton et al., using a cine true FISP sequence, compared functional and morphological cardiac evaluations at 3T and at 1.5T [50]. The CNR at 3T, using a much smaller flip angle in order to avoid SAR limitations, was similar to the one obtained in the 1.5T scans. Susceptibility artifacts were also increased using the b-SSFP sequence. In order to avoid the potential banding artifact, a short TR and per-patient shimming is advised [51]. ECG triggering was acceptable at both field strengths, based on no increased motion artifacts in the images when comparing 1.5T to 3T.

SNR increase comes with the penalty of an increase in the artifact-to-noise ratio (ANR). Artifacts that were already present at 1.5T become more prominent at 3T, such as errors in parallel imaging and a deficient crusher gradient [51]. An increase of CNR in the endocardial boundary would tend to make Gibbs ringing and motion artifacts more visible, but the high SNR enables faster imaging and reduced motion artifact. Chemical shift artifacts also increase at 3T, but can be alleviated by increasing the bandwidth with an SNR penalty.

Another current problem with high-field systems is the fact that imaging parameters, RF pulse profiles, and RF coil designs have been extensively optimized at 1.5T whereas 3T optimization is now only being performed[51,52] As 3T scanners gain a wider market share, this concern will most likely disappear. With an optimized system, the values of increased SNR and CNR can be utilized, making high-field systems a contender for cardiovascular MRI.

### Imaging protocols & indications

#### Imaging indications

Currently, the two major clinical indications for use of MR perfusion imaging are: 1) the detection and sizing of microvascular obstruction in patients after acute myocardial infarction, and 2) the detection of ischemia in patients with suspected coronary artery disease. In both cases, perfusion imaging pulse sequences will likely be used in combination with other pulse sequences, such as segmented k-space cine sequences to allow assessment of myocardial function, and inversion recovery sequences to allow assessment of myocardial viability. However, since the clinical objectives of CMR in these two groups of patients are different, most investigators will use different study protocols for each type of patient. Also, the image parameters for each group of patients will likely differ to accommodate for the different requirements in these two clinical situations.

#### Evaluation of patients with acute myocardial infarction

**Figure 4:**
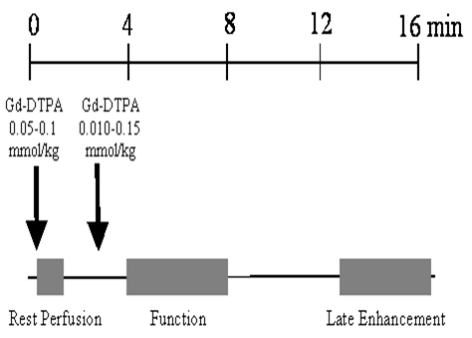
Typical protocol for assessing extent of microvascular obstruction with rest first-pass perfusion imaging. An alternate method of imaging microvascular obstruction is single shot late gadolinium enhancement.

In patients with acute MI, the main purpose of myocardial perfusion imaging is the precise estimation of the size of microvascular obstruction (no-reflow area),[53] if present. A typical protocol, which might be used for assessment of microvascular obstruction in patients with acute MI, is illustrated in Figure 4. Resting perfusion imaging is performed during bolus injection (3–4 ml/min) of a low dose (0.05–0.1 mmol/kg) of Gd chelate. Since the purpose of this resting perfusion study is accurate sizing of the area of microvascular obstruction/no-reflow, one must increase the spatial coverage and compromise temporal resolution. Since the perfusion defects due to microvascular obstruction fill in relatively slowly, temporal resolution is less important than for stress perfusion studies. Typically, dynamic imaging with full coverage of the entire heart, e. g., 8–10 short axis slices, is performed with a repetition rate every 2 or 3 heart beats. After the completion of the rest perfusion study, additional contrast can be infused for a total dose of 0.15–0.2 mmol/kg for subsequent LGE CMR images for infarct sizing. These LGE images are acquired 10–20 minutes after the contrast injection, with inversion recovery gradient pulse sequences. An alternative approach is to just obtain single shot LGE images to visualize microvascular obstruction. However, the time between first-pass perfusion sequence and LGE images can be used to acquire functional SSFP cine images. Since image contrast in SSFP is determined by the T2/T1 ratio of the tissue, SSFP image quality is often not significantly impaired.

#### Evaluation of patients with suspected coronary artery disease

Suspected coronary artery disease can be most dramatically demonstrated during stress-induced ischemia on MR perfusion imaging performed during maximal coronary vasodilation. Vasodilatation can be induced by infusion of either dipyridamole or adenosine. Both agents act through stimulation of A2 receptors in the microvasculature. Adenosine is the direct agonist of this receptor. On the other hand, dipyridamole is a pro-drug, whose liver metabolite exerts its effect by an indirect mechanism on the endothelium – blocking the cellular reuptake of adenosine and inhibition of adenosine deaminase in endothelial cells, which leads to increased extracellular concentrations of adenosine. The stimulation of the A2 receptors relaxes the resistive arterioles, which normally autoregulate myocardial perfusion relative to coronary perfusion pressure. In normal non-ischemic myocardium, this results in maximal hyperemic vasodilation with an increase of myocardial perfusion without changes in myocardial blood volume [54]. In myocardium subtended by a signficant epicardial coronary stenosis, the magnitude of the perfusion increase during vasodilation is compromised compared to normal myocardium, because of the drop of coronary perfusion pressure downstream of the coronary stenosis [54]. This pressure drop results in capillary closure, reduced perfusion, and reduced blood volume during hyperemia, which all translates into a slower arrival and lower contrast agent concentration in the ischemic as compared to normal myocardium. Thus, the ischemic regions demonstrate reduced signal intensity relative to the normal myocardium on T1-weighted images during vasodilation and appear as a perfusion defect [54].

Two different protocols are currently used for the infusion of dipyridamole. The low-dose injection protocol consists of an infusion of a total dose of 0.56 mg/kg dipyridamole in 4 minutes, with imaging started immediately after completion of the 4 minute infusion. The high-dose injection protocol adds a second injection of 0.28 mg/kg dypyridamole for 2 minutes duration, commencing at 8 minutes after the beginning of the first injection. Thus, a total dose of 0.84 mg/kg dipyridamole is administrated in this high-dose dipyridamole infusion protocol. Imaging is initiated at the end of the second infusion, i.e., 10 minutes after the beginning of the first dipyridamole infusion. The advantage of the high-dose dipyridamole infusion protocol is a more intense, longer lasting and more predictive vasodilatation response than that obtained by the low-dose protocol.

For the adenosine infusion protocol, adenosine is injected at a rate of 0.14 mg/kg/min, typically for 3–6 minutes for a total dose of 0.48 to 0.84 mg/kg, with imaging during the terminal portion of the adenosine infusion. Adenosine and contrast are administered via separate IVs to avoid large bolus drug delivery that increases risk of heart block. The adenosine infusion can be stopped when the contrast is completed. Both dipyridamole and adenosine can cause similar side effects, which may include light flushing, mild chest discomfort, and headache. Patients with significant coronary artery disease may experience angina pectoris. More serious side effects include atrioventricular (AV) conduction block, bronchospasm or cerebral hypoperfusion with potential neurological symptoms in the presence of significant carotid stenosis.

The advantage of dipyridamole over adenosine is the lower cost and longer duration of vasodilation with a half-life of ~30 minutes versus < 10 seconds for adenosine, which gives a longer time period for performing the perfusion imaging. This longer half-life of dipyridamole can be associated with a longer duration of potential side effects that are not as self-limiting as adenosine. On the other hand, the short half-life of adenosine may give less time to respond to potential side effects. Because of the potential side effects, patients need to be monitored before, during, and after stress protocols, and equipment and qualified personnel for life support and resuscitation needs to be available. Since the vasodilating effects of both dipyridamole and adenosine can be abolished by ingestion of xanthine inhibitors, patients should withhold caffeinated beverages and theophylline for 24 hours prior to imaging [55]. Dipyridamole and adenosine are contraindicated in patients with severe conduction alterations, obstructive pulmonary disease (COPD and asthma) and severe carotid stenosis. Neither drug should be administered to pregnant women.

After induction of vasodilation by either adenosine or dipyridamole, first-pass perfusion imaging is performed during intravenous bolus injection of a gadolinium-based contrast agent. The objective of the test is the detection of perfusion defects in the ischemic area. Due to the extravascular distribution of most clinically approved contrast agents, such perfusion defects persist very briefly after bolus injection, before disappearing due to redistribution into the extravascular space. Hence, stress MR perfusion imaging requires very rapid image acquisitions. The focus of the pulse sequence will thus be maximal temporal resolution, at the cost of left ventricular coverage and spatial resolution. The best available perfusion sequences typically allow 3–4 short and/or long-axis slices to be acquired in a dynamic mode with a temporal repetition rate of *every *heart beat.

**Figure 5:**
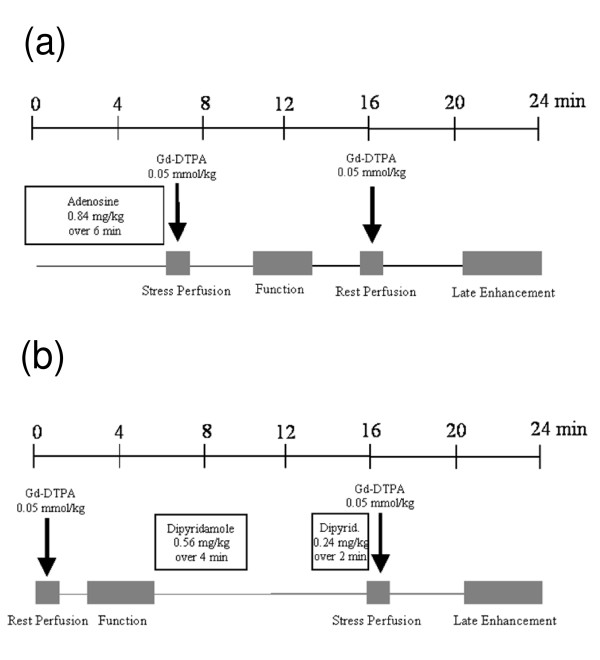
(A) Typical first-pass perfusion protocol for adenosine stress perfusion CMR. (B) Typical first- pass perfusion protocol for dipyridamole stress perfusion imaging.

Typical MR stress perfusion protocols are illustrated in Figure 5. Most protocols consist of stress and rest perfusion studies, to allow distinction of perfusion defects from ischemia versus image artifacts. The optimal order of rest and stress perfusion test may be different for adenosine versus dipyridamole protocols. Due to the short half-life of adenosine, it may be better to perform the stress study first so that the stress perfusion measurement is not contaminated by residual gadolinium injected at rest. Indeed, if resting perfusion is performed first, there is a potential risk that Gd might accumulate in infarcted areas and mask the inducible ischemic areas in a subsequent stress test. However, in the case of dipyridamole, it may be better to first perform the resting study and then the stress study, since the long-lasting vasodilatory effect of dipyridamole can persist and delay the rest study by more than 15 minutes. Thus, adenosine may be the preferred vasodilator for MR perfusion studies.

### Staffing and technical components required for patient stress testing

Patient safety during myocardial perfusion assessment requires appropriate staffing and technical considerations. The three types of staff typically involved in performing a stress CMR examination include technologist, nurse, and physician. The nurse and physician should be certified in basic and advanced cardiac life support with experience in anticipating, recognizing, and managing cardiac events that may occur secondary to or independently of pharmaceutical-induced effects. This is facilitated by having MR-compatible monitoring equipment in the scanner room with an additional display outside the room if needed. The minimum monitoring needs include continuous electrocardiographic display and heart rate measurement as well as periodic blood pressure recording. Capabilities for noninvasive oximetry are often helpful, particularly for the patient with obstructive lung disease of uncertain severity undergoing stress with vasodilators that may produce bronchoconstrictive effects. Weight-based vasodilator doses for stress are typically prepared by the pharmacy staff and delivered to the MR lab at the start of the procedure. In the MR lab area, there is also a point-of-care automated medication storage unit accessible by the clinical staff that is stocked with medications such as aminophylline, metoprolol, and lidocaine that may be needed for acute reversal or therapy of complications. In instances requiring defibrillation or resuscitation, there should be a plan in place that clearly delineates individual responsibilities when a patient needs to be brought out of the scanner room. One individual is typically responsible for patient transferal, another goes to get the resuscitation equipment or "crash cart", and the physician is responsible for directing appropriate inteventions. Such events may trigger the arrival of many individuals, who arrive to help with resuscitation but may not be facile with principles of MR safety. Thus, vigilance by the MR staff is required.

### Pre- and post-procedure patient assessment

Prior to the exam, the nurse typically screens the patient for clinical history that would preclude administration of adenosine or dipyridamole. This is done in conjunction with screening for MR contraindications, such as presence of ferromagnetic foreign body, aneurysm clip, intraorbital metal, or non-MR compatible implant. As noted earlier, a history of obstructive airway disease or conduction abnormalities should prompt the discussion of whether adenosine or dipyridamole stress testing is appropriate. A baseline electrocardiogram (ECG) is acquired prior to the MRI examination.

After the exam, the patient is returned to the preparatory/recover area outside the scanner room where the nurse repeats a 12-lead ECG. The yield of this tracing is fairly low, given the rarity of ST changes due to adenosine. However, ischemic ECG changes resulting from adenosine portend a high likelihood of significant obstructive coronary artery disease. A post-procedure blood pressure measurement should also be documented, particularly if there was any significant blood pressure deviation during the exam.

### Image analysis

#### Qualitative interpretation

**Figure 6:**
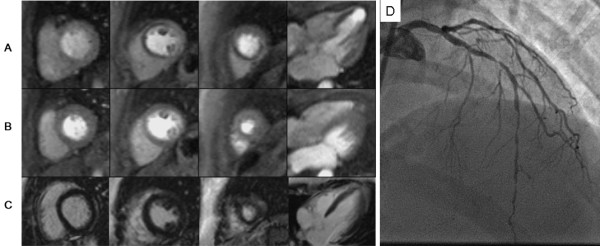
Myocardial perfusion CMR was performed in a 43 year-old female to evaluate exertional chest pressure. Perfusion images obtained during intravenous infusion of adenosine (A) demonstrate severe hypoenhancement of the septum, anterior wall, and apex that is not present on resting perfusion imaging (B). Late gadolinium enhancement acquisitions are negative for hyperenhancement (C); together, these findings suggest severe myocardial ischemia in the distribution of the left anterior descending coronary artery without infarction. (D) Invasive coronary angiography confirms high-grade serial stenoses in the left anterior descending coronary artery. (See Additional file 2.)

**Figure 7:**
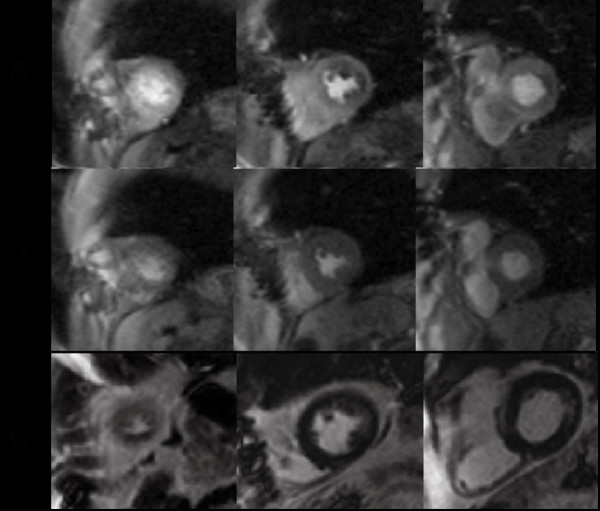
First-pass myocardial perfusion CMR was performed in a 75 year-old male with a history of bypass surgery seeking a second opinion regarding intervention for bypass graft disease. Perfusion images obtained during intravenous infusion of adenosine (A) demonstrates diffuse subendocardial perfusion abnormality not present on resting perfusion imaging (B). Late gadolinium enhancement acquisitions (C) show a small region of enhancement involving the lateral wall. Together, these findings suggested prior infarct with ischemia. His symptoms were controlled with optimization of anti-anginal medical therapies. (See Additional file 3.)

Qualitative interpretation of perfusion CMR images remains the mainstay of clinical reporting in 2007. Furthermore, stress perfusion interpretation almost always requires concomitant acquisition and interpretation of LGE images. A step-wise algorithm that begins with review of LGE images, followed by stress and rest perfusion images works well for overall classification of presence or absence of epicardial coronary disease in most cases [46]. After carefully identifying the presence and extent of infarct scar on LGE imaging, the interpreting physician can better determine whether perfusion defects match regions of scar vs. those that extend beyond the scar. In the absence of scar or artifact (see below), uniform myocardial enhancement throughout the myocardium in all slices at rest and with vasodilator stress indicates absence of ischemia. With LGE in a region and corresponding defect on stress perfusion images, the interpretation of "fixed defect, no ischemia" is appropriate. When a region demonstrates stress perfusion abnormality that normalizes at rest without LGE, the interpretation of "positive for ischemia" follows, barring artifact (Figure 6). A region of perfusion abnormality with stress that corresponds to the scar on LGE but subtends a greater myocardial extent warrants description as "infarct with peri-infarct ischemia." Clinically, this is helpful to the referring physician when judging whether or not to revascularize a chronically occluded vessel (Figure 7). Viable myocardium based on transmural extent of scar along with extent of ischemia may help guide assessment of the need for and likely response to revascularization in such cases [56]. Finally, some patients undergoing stress CMR may have circumferential subendocardial ischemia. Angiography may be required to classify diffuse subendocardial perfusion abnormality with vasodilator stress as multivessel or left mainstem coronary artery disease versus microvascular disease, as has been described in patients with syndrome X [57].

#### Quantification and validation

##### Image analysis for quantitative MRI perfusion studies

In analyzing first-pass contrast enhancement images for quantitative assessment of cardiac perfusion, we need to be able to position a region of interest (ROI) within the wall, in order to follow the dynamic changes in MRI signal intensity. While in principle this is straightforward, there are some relevant issues that may need to be considered. First, we need to position the ROI carefully, so as to avoid contaminating the wall signal with signal from the adjacent blood. Another potential problem with ROI positioning can arise if there are premature ventricular beats, leading to variability of the size of the ventricular cavity and thus the position of the wall. Also, since patients are often unable to hold their breath for the full duration of the sequence of dynamic enhancement images, interactively repositioning of the ROI on sequential images may be needed to track a consistent portion of the heart wall.

One frequent method of segmentation is the 17-segment model proposed by the American Heart Association, corresponding to the typical blood flow distributions of the coronary arteries [58]. The epicardial and endocardial borders of the heart are defined, and then the resulting annular regions are divided into suitable septal and free wall segments. As the subendocardial portion of the heart wall is most vulnerable to ischemia, it can be useful to further subdivide the segmental ROIs into endocardial and epicardial halves.

As there is no absolute scale of image intensity in MR, and there are many potential sources of variability of the image intensity, an approach to standardize and reproducibly quantify dynamic intensity changes is needed. One approach is to acquire additional calibration images, whose intensities must also be measured. For example, acquiring a "proton density" image at the same location can be used both to provide an "absolute" intensity scale and to correct for shading due to the use of local surface coils [59,60]. A separate noise image can also be acquired for estimating possible noise bias in low intensity images (as may be seen prior to the arrival of the contrast bolus in the T1-weighted images).

In any attempt at quantitative analysis of dynamic first-pass perfusion images, we need to be able to account for the effect of potential variations in the rate, amount, and timing of the delivery of the contrast agent in the blood (the arterial input function) on the resulting enhancement of the heart wall. One way to estimate the arterial input function (AIF) is to position an ROI within the left ventricular cavity, while minimizing contamination due to volume averaging, and follow the sequential signal changes in the blood.

##### Quantification of CMR perfusion

There are many potential problems associated with the determination of contrast concentrations from serial MR intensity measurements. First, the relationship between contrast concentration and signal is nonlinear, and also depends in a nonlinear way on the imaging parameters, such as the time between the saturation pulse that produces the T1 weighting and the image data acquisition. This nonlinearity can be particularly problematic when the concentration of contrast agent is high, as during the peak enhancement phase, as the response of the signal intensity may be effectively saturated, leading to a tendency to underestimate the concentration. Regional variations in the RF excitation field (B1) and the main magnetic field of the MR system (B0) can produce a corresponding variation of the amount of residual magnetization left after the nominal saturation pulse, with a resulting uncertainty of the amount of the contrast agent-induced signal increase [21]. Finally, there may be some uncertainty as to whether it is reasonable to assume that all the water in the tissue is equally exposed to the contrast agent ("fast exchange" condition) [61].

Assuming that we can determine the time course of the contrast agent concentrations in the arterial blood and the myocardium, calculation of the corresponding perfusion blood flow and related parameters can then be done. A hypothetical instantaneous bolus input ("impulse") of contrast agent at the tissue arteriolar level would produce a corresponding transient increase in the tissue concentration that would depend on the blood flow into the tissue, and would last for at least the duration of the transit of the labeled blood through the tissue. The mean transit time (MTT) of an instantaneous bolus is given by the ratio of the fractional blood volume of the tissue and the flow. As conventional contrast agents can diffuse from the vascular space into the extravascular space (but not into normal cells), the observed transit will be longer than the nominal vascular MTT. Obviously, any delay or prolongation of the delivery of the contrast agent to the tissue will be directly reflected in the observed tissue response, effectively being combined ("convolved") with the ideal impulse response, and will also need to be accounted for.

A variety of approaches have been proposed to approximately correct the observed tissue enhancement for the effects of the AIF. These include calculating various parameters of the observed tissue enhancement curves, such as the time of arrival of the contrast agent, the time of the peak enhancement, and the slope of the enhancement curve, and correcting them by subtracting or scaling by the corresponding AIF parameters. The presence of noise in the data and other experimental limitations can lead to problems in reliably defining these parameters in practice. Furthermore, these empirical estimates don't allow a full calculation of the tissue transit effects or extravascular exchange of the contrast agent. A potentially better approach is to approximately mathematically undo ("deconvolve") the effects of the AIF from the observed tissue response, in order to estimate the corresponding underlying MTT and calculate the blood flow; a variety of approaches have been proposed for carrying out such calculations [29,62,63].

In light of the numerous obstacles outlined above, no quantitative perfusion analysis technique has gained widespread clinical use at this time. However, in research endeavors and in some clinical settings, one may use a relative index of myocardial perfusion reserve [7,57,64,65]. For example, using semiautomatic delineation of endocardial and epicardial left ventricular borders throughout the phases of first-pass perfusion, with respiratory motion correction as needed, rest and stress myocardial perfusion slopes may be derived using Fermi-fitting of signal intensity versus time and normalized to the LV blood pool slope as well as heart rate [57]. A Myocardial Perfusion Reserve Index (MPRI) is then the ratio of stress to rest normalized myocardial perfusion slopes.

### Clinical interpretation of first-pass CMR perfusion

Cardiovascular MR perfusion imaging with rest and stress acquisitions also helps answer a variety of clinical questions in the subacute or chronic setting. For example, the patient with atypical chest pain in whom ischemic heart disease is suspected may undergo rest/stress CMR perfusion imaging with LGE to define the presence or absence of flow-limiting coronary artery disease as the cause of symptoms. This is particularly helpful if there is known coronary artery disease and prior infarction. The superiority of CMR to delineate infarct scar becomes critical in providing this information in addition to extent of ischemia [66]. In patients identified as having anatomic lesions of uncertain hemodynamic significance by angiography, CMR with rest/stress perfusion may be helpful to define the angiographic lesion(s) producing impaired myocardial blood flow under stress conditions.

### Rest perfusion CMR in acute MI patients

Detection of microvascular obstruction can assist in the distinction of chronic from acute infarcts, since microvascular obstruction occurs exclusively in acute myocardial infarction and disappears progressively over time [67]. The presence of microvascular obstruction in an infarcted area can be a marker of non-viable tissue in which function rarely recovers [68-72]. More importantly the presence of microvascular obstruction has been shown to have negative prognostic value in patients with acute infarcts [67,73,74]. Patients who had infarcts with microvascular obstruction were found to have significantly more events, such as death and hospitalization for heart failure, and increased adverse remodeling [73-75].

### Stress perfusion CMR in patients with suspected coronary artery disease

#### Diagnosis of coronary artery disease in with stable chest pain

The results of nine studies of 618 patients, which reported diagnostic accuracy on per-patient basis for detection of coronary artery disease versus detection of invasive coronary angiography, are summarized in Table 1. Overall, in these studies, perfusion CMR had high sensitivity (89%), specificity (81%), and accuracy (86%) for detection of significant (> 75% diameter stenosis) coronary artery disease on a per-patient basis. Similarly high accuracy was reported in other single-center studies[64,76,77] on a segmental basis alone.

**Table 1:** Diagnostic accuracy of perfusion MR for assessment of coronary artery disease

**Author**	**Remarks**	**# of patients**	**Prevalence of CAD**	**Sensitivity (%)**	**Specificity (%)**	**Accuracy (%)**
Schwitter et al. [7]	upslope	57	65%	86%	85%	86%
Nagel et al. [90]	upslope	84	51%	88%	90%	89%
Ishida et al. [82]		104	74%	90%	85%	88%
Wolff et al. [78]	low dose group	26	54%	93%	75%	85%
Giang et al. [79]	group 2 and 3	44	64%	93%	75%	86%
Paetsch et al. [83]	upslope	79	67%	91%	62%	81%
Plein et al. [91]	upslope	92	64%	88%	82%	86%
Sakuma et al. [8]	visual	40	53%	81%	68%	75%
Klem et al. [46]	visual: perfusion and late enhancement	92	40%	89%	87%	88%

At present, two multicenter studies have examined the influence of contrast agent dose on diagnostic accuracy. [78,79] Most studies have examined the diagnostic accuracy for detection of anatomical coronary artery stenosis (defined either as > 50% or > 75% luminal diameter reduction). The limitation of this approach is that functional severity of a coronary stenosis may vary and depend on parameters other than lumen diameter. Two studies [7,80] compared flow reserve by CMR versus PET, the non-invasive gold-standard at present, and demonstrated a high correlation and diagnostic accuracy. In another study, Rieber et al.[81] compared the diagnostic accuracy of perfusion CMR versus angiographically measured fractional flow reserve and reported good diagnostic accuracy of perfusion CMR to this invasive gold-standard.

A few studies have compared diagnostic accuracy of CMR versus other techniques for detection of coronary artery disease. Ishida et al.[82] showed a higher diagnostic accuracy of perfusion CMR than SPECT for detection of significant CAD. Similarly, Sakuma at al.[8] observed a similar higher diagnostic accuracy for CMR than for 201Tl SPECT. Paetch et al.[83] reported similarly high sensitivity, specificity, and accuracy compared to dobutamine stress MR for detection of coronary artery disease.

#### Patients with chest pain with suspected unstable coronary artery disease presenting to the emergency room

In a study of 135 patients, who presented to the emergency department with chest pain, but without acute myocardial infarction, Ingkanisorn et al.[84] reported that an abnormal adenosine perfusion CMR exam had a 100% sensitivity and 93% specificity for predicting future events, such as coronary artery disease, new MI, or death. This suggests that perfusion CMR could have a potential role in selecting patients with high risk in the emergency department.

#### Perfusion CMR in other diseases

Panting et al.[57] employed perfusion CMR to elucidate the cause of chest pain in patients without significant coronary artery disease or Syndrome X. A reduced flow reserve was shown in the subendocardium, but not in the subepicardium in these patients, suggesting a potential ischemic origin for Syndrome X. A study by Vermeltfoort et al.[85] did not confirm this finding, but a more recent study by Lanza et al. was confirmative and showed Doppler derived coronary flow abnormalities in the abnormal regions identified by perfusion CMR [86]. In addition, abnormalities in subendocardial perfusion have been recently reported in patients with repaired aortic coarctation, a population that suffers early death due to myocardial ischemia [87].

### Prognostic value of stress perfusion CMR

Finally, perfusion CMR has also been shown to have predictive value in patients with coronary artery disease. Indeed, Jahnke et al.^79 ^evaluated the prognostic value of this technique in 533 patients with chest pain or dyspnea and suspected coronary artery disease in comparison to dobutamine stress MR. Over a median follow-up of 2.3 years, ischemia by perfusion CMR was found to be the single best predictor of cardiac death or nonfatal myocardial infarctions and was superior to ischemia on dobutamine stress CMR. Presence of a normal perfusion test was predictive of 99% chance of a 3 year event-free survival.

### LGE/stress/rest perfusion vs. other approaches

It should be remembered that clinicians have choices among several modalities when deciding whether or not to refer individual patients for perfusion CMR examination, particularly stress perfusion imaging. The value added by LGE imaging comes from its proven reliability in delineating extent of infarcted myocardium[56] as well as its superior ability to detect myocardial disease due to other nonischemic cardiomyopathies [88]. Incorporating other measurements, such as presence of significant valvular disease and velocity-encoded data to assess diastolic function[89]^1^, allows a brief, single CMR exam to provide comprehensive assessment for etiologies of cardiovascular signs and symptoms that might otherwise require multiple testing and, therefore, less cost-effective healthcare.

## Competing interests

The authors declare that they have no competing interests.

## Supplementary Material

Additional file 1 Gibbs artifact. This cine shows signal intensity profile resulting in dark rim artifact caused by Gibbs ringing. Click here for file

Additional file 2 LAD-territory ischemia with adenosine stress perfusion. This cine shows myocardial ischemia in the distribution of the left anterior descending coronary artery. Stress perfusion images in basal, mid and apical short axis planes as well as a horizontal long axis plane are shown across the top, and rest perfusion images in the same planes are shown across the bottom. Ischemia appears as dark regions (hypoperfusion) in the stress images. Click here for file

Additional file 3 Subendocardial ischemia with adenosine stress perfusion. This cine shows diffuse subendocardial perfusion abnormality, most prominent along the lateral wall, with adenosine stress. Stress perfusion images in apical, mid and basal short axis planes are shown across the top and rest perfusion images in the same planes are shown across the bottom. Ischemia appears as dark regions (hypoperfusion) in the stress images. Click here for file
